# KLOSURE: Closing in on open–ended patient questionnaires with text mining

**DOI:** 10.1186/s13326-019-0215-3

**Published:** 2019-11-12

**Authors:** Irena Spasić, David Owen, Andrew Smith, Kate Button

**Affiliations:** 1 School of Computer Science & Informatics, Cardiff University, Cardiff, UK; 2 School of Psychology, Cardiff University, Cardiff, UK; 3 School of Healthcare Sciences, Cardiff University, Cardiff, UK

**Keywords:** Text mining, Natural language processing, Text classification, Named entity recognition, Sentiment analysis, Patient reported outcome measure, Open-ended questionnaire

## Abstract

**Background:**

Knee injury and Osteoarthritis Outcome Score (KOOS) is an instrument used to quantify patients’ perceptions about their knee condition and associated problems. It is administered as a 42-item closed-ended questionnaire in which patients are asked to self-assess five outcomes: pain, other symptoms, activities of daily living, sport and recreation activities, and quality of life. We developed KLOG as a 10-item open-ended version of the KOOS questionnaire in an attempt to obtain deeper insight into patients’ opinions including their unmet needs. However, the open–ended nature of the questionnaire incurs analytical overhead associated with the interpretation of responses. The goal of this study was to automate such analysis. We implemented KLOSURE as a system for mining free–text responses to the KLOG questionnaire. It consists of two subsystems, one concerned with feature extraction and the other one concerned with classification of feature vectors. Feature extraction is performed by a set of four modules whose main functionalities are linguistic pre-processing, sentiment analysis, named entity recognition and lexicon lookup respectively. Outputs produced by each module are combined into feature vectors. The structure of feature vectors will vary across the KLOG questions. Finally, Weka, a machine learning workbench, was used for classification of feature vectors.

**Results:**

The precision of the system varied between 62.8 and 95.3%, whereas the recall varied from 58.3 to 87.6% across the 10 questions. The overall performance in terms of F–measure varied between 59.0 and 91.3% with an average of 74.4% and a standard deviation of 8.8.

**Conclusions:**

We demonstrated the feasibility of mining open-ended patient questionnaires. By automatically mapping free text answers onto a Likert scale, we can effectively measure the progress of rehabilitation over time. In comparison to traditional closed-ended questionnaires, our approach offers much richer information that can be utilised to support clinical decision making. In conclusion, we demonstrated how text mining can be used to combine the benefits of qualitative and quantitative analysis of patient experiences.

## Background

Musculoskeletal pain is recognised globally as negatively impacting healthy aging and accounts for 21.3% of total years lived with disability [1]. It is associated with frailty, loss of function and independence during everyday activities and reduced overall physical and mental wellbeing [2]. The knee is one of the most commonly affected joints reportedly affecting 30% of people with joint pain [3]. Knee osteoarthritis is one of the most common conditions and affects 18% of people over the age of 45 in England [4]. With an aging population, this number is set to increase, placing greater burden on health resources [4], adding to waiting lists and causing delays in receiving appropriate care [5]. Therefore, self-management treatment approaches, which equip patients with the skills to manage their health condition, are essential. When away from the secondary care setting, patient reported outcome measures (PROMs) can be used to monitor their health status remotely.

PROMs are standardised, validated questionnaires completed by patients in an attempt to measure their own perceptions of their health conditions. Patient responses are converted into a numerical score, which can be used to monitor patient progress over time and plan treatment accordingly. The Knee injuries and Osteoarthritis Outcome Score (KOOS) [6] is one of the most widely used PROMs for assessing patients’ opinions about their knee condition. It is administered as a 42-item closed-ended questionnaire in which patients are asked to assess five outcomes: pain, other symptoms, activities of daily living, sport and recreation activities, and quality of life. The resulting scores on a scale of 0–100 can help both patients and clinicians to monitor the progress of knee rehabilitation. However, KOOS does not capture details surrounding particular patient circumstances [7]. By forcing the respondents to choose from ready-made options, closed-ended questions restrict freedom and spontaneity of responses and as such they are unlikely to tap into the full range of positive and negative expressions of patients [8]. Alternatively, modifying KOOS into an open-ended questionnaire has got a great potential to inform clinicians about patients’ opinions including their unmet needs, but this incurs analytical overhead associated with the interpretation of responses.

Unfortunately, patient experience questionnaires remain largely quantitative in nature [9] despite the findings that they tend to overestimate patient satisfaction [10] and that qualitative analysis tends to uncover more actionable information [11]. This can be explained partly by the lack of knowledge on how best to collect and present patient’s responses to the stakeholders [10]. From a practical point of view, the cost of qualitative analysis in terms of time and labour play a major factor in its prevalence or the scale of such studies. Poorer statistical significance has often been used as an excuse to dismiss valuable information that can be provided by qualitative research [12]. In light of these issues, text mining (TM), which aims to discover patterns, relationships and trends within text documents, has found a great many biomedical applications [13]. In particular, it is increasingly used to analyse patient experiences, i.e. their thoughts, feelings and behaviours, expressed in their own words [14]. Therefore, TM may support scalability of qualitative analyses of open-ended questionnaires.

To date, most TM approaches used to support the analyses of open-ended questionnaires focused on aggregating all responses across a surveyed population, e.g. consumers [15], students [16, 17], patients [18], etc. Early techniques used to process open-ended questionnaires included rule-based text classification approaches [15]. The proliferation of user-generated data on the Web encouraged the use of data mining, e.g. clustering [16] and association rule mining [19]. The rising popularity of supervised machine learning approaches paved the way to classifying individual responses. In terms of the overall aim and techniques applied, the work on screening patients for posttraumatic stress disorder is the closest to our own [20]. They used an open-ended questionnaire to elicit self-narratives from participants who experienced a traumatic event. They used supervised machine learning to implement a binary classifier with an aim to automatically diagnose a participant, based on their overall response, as having or not having posttraumatic stress disorder. Our approach goes a step further by classifying a response to each open-ended question separately against multiple classes.

## Methods

The aim of this study was to automate measurement of health outcomes from patients’ free–text responses to open–ended questions. Addressing this aim required us to: (1) develop an open–ended questionnaire, (2) collect responses to the questionnaire, (3) analyse the responses manually to establish the ground truth, (4) develop text mining methods to analyse the responses automatically, and (5) evaluate the performance of the text mining methods. The following sections describe these steps in more detail.

### Open-ended questionnaire

#### Questionnaire development

We developed an open–ended questionnaire to capture patients’ opinions about all aspects relevant to assessing the management of their knee condition while minimising the number and complexity of questions. We designed KLOG (contracted from ‘knee log’) as an open–ended version of the KOOS questionnaire [6]. Like KOOS, KLOG was designed to elicit responses that can be used to assess five outcomes: pain, other symptoms, activities of daily living, sport and recreation, and quality of life. The open–ended nature of the questionnaire enabled us to reduce the number of questions from 42 in KOOS to only 10 in KLOG. For example, KOOS contains 9 closed–ended questions related to pain: (P1) How often do you experience knee pain? What amount of knee pain have you experienced the last week during the following activities? (P2) twisting/pivoting on your knee, (P3) straightening knee fully, (P4) bending knee fully, (P5) walking on flat surface, (P6) going up or down stairs, (P7) at night while in bed, (P8) sitting or lying, (P9) standing upright. KLOG compresses them into a single open–ended question: *“Can you describe any knee pain you have experienced over the past week?”* To illustrate a greater coverage of the open-ended question, we provide a sample of answers:

1. <P1>*Constant*</P1> *pain whether in* < P8>*sitting*</P8> *or* < P9>*standing*</P9>*.*
2. <P1>*Occasional*</P1> *sharp pain as well as ache, especially* < NA>*walking downhill*</NA> *or on* < NA>*uneven ground*</NA>*.*
3. *No pain when* < P8>*sitting*</P8>*, some pain when* < P6>*walking upstairs*</P6> *and* < NA>*walking long distances*</NA>*.*
4. <P1>*Occasional*</P1> *sharp pain especially when* < P6>*going up steps*</P6>*.*
5. *Some general pain when* < NA>*exercising*</NA> *and quite painful in the joint for* < P1>*the last 2 days*</P1>*.*
6. *I have severe aching and bad pain when I* < NA>*get up from sitting*</NA> *and* < P5>*general walking*</P5>*.*
7. <P1>*Occasionally*</P1> *the knee joint aches together with momentary sudden stabbing pain in various parts of the knee. Also, pain if I am* < P8>*sat*</P8> *with the knee* < P4>*bent*</P4> *such as* < P8>*sat*</P8> *in an office chair.*
8. <NA>*Exercise*</NA> *induced occasional medial and lateral knee joint discomfort knee. Plus some* < P7>*nocturnal*</P7> *discomfort.*

We used XML tags to relate free–text answers to the corresponding questions in KOOS as shown in the above answers. We can see that when answering a more generic open–ended KLOG question, patients do provide information related to the corresponding closed–ended questions in KOOS, but they also provide a much richer account of circumstances surrounding their experience of pain. We annotated such references using the NA tag to indicate that the corresponding closed–ended question is not available in KOOS.

To pre–test the KLOG questionnaire, we administered it to a small number of knee patients (16 in total). Participants were asked to complete the questionnaire on a weekly basis over the course of 4 weeks. We obtained a total of 30 responses. The size of the dataset was 4625 words, which is equivalent to 10.3 A4 pages using Arial 12 pt. and single spacing. We analysed the data qualitatively to identify potential issues with the questionnaire. Based on the results, the original questions were re–phrased and re–ordered where appropriate. For example, the original question *“Can you describe any knee symptoms you have experienced over the past week?”* was re–phrased to *“Can you describe any knee symptoms other than stiffness and pain you have experienced over the past week?”* and moved after the questions about stiffness and pain to prevent overlapping answers.

#### Online questionnaire administration

To collect answers remotely, we implemented a web site using a responsive design for user–friendly access on a range of Internet–enabled devices. In the online version of the KLOG questionnaire, users were asked to answer a series of 10 questions in a fixed order:

1. What knee condition are you currently receiving treatment for?
2. What treatment are you currently using for your knee?
3. Have there been any changes to your knee condition over the past week?
4. How confident do you feel about looking after your knee?
5. Can you describe any knee stiffness you have experienced over the past week?
6. Can you describe any knee pain you have experienced over the past week?
7. Can you describe any knee symptoms other than stiffness and pain you have experienced over the past week?
8. Can you describe if your knee condition limited your ability to carry out your day to day tasks over the past week?
9. Can you describe if your knee condition limited your ability to carry out your work, hobbies or exercise over the past week?
10. Do you have any other comments about your knee condition or the treatment you are receiving?

All questions but the last were mandatory. A progress bar was used to visualize the progression through the questionnaire in an attempt to increase completion rates. The access to the questionnaire was gated through a consent form and participant information sheet. All data collected online was stored securely in a back–end database.

#### Questionnaire response analysis

To automatically process patients’ responses to the KLOG questionnaire, we implemented a text mining system, KLOSURE (contracted from ‘KLOG measure’). The analysis is performed for each question independently. To interpret answers to questions about the knee condition (Q1) and its treatment (Q2), the system aims to recognise relevant named entities (NEs). For example, to identify what condition a patient is treated for, the system looks for mentions of *medical conditions* (NE1) or *surgical procedures* (NE2) as well as mentions of affected *anatomical structures* (NE3), e.g.

*I <* ne1*>completely ruptured</*ne1*> my <* ne3*>ACL</* ne3*> and had <* ne3*>ACL</* ne3*> <* ne2*>reconstruction surgery</*ne2*> using a <* ne2*>hamstring graft</*ne2*>.*

In addition to finding NEs, the system maps them onto the Unified Medical Language System (UMLS) [21], the largest curated collection of biomedical entities. Such mapping normalises representation of NEs by associating all synonyms with a unique identifier. This fact enables text data to be managed, searched and processed based on their meaning. In KLOSURE, named entity recognition (NER) is used not only to provide the final output in relation to questions Q1–Q2, but also to extract NEs as auxiliary features in relation to the remaining questions. The same NER approach is used in both cases. Therefore, to simplify description of the system, we hereby focus on processing answers to questions Q3–Q10 as the main functionality of the system.

KLOSURE aims to classify free–text answers to questions Q3–Q10 on a 3–point Likert scale. This task is known as ordinal classification, where the rating of a data item is estimated on a fixed, discrete rating scale. Ordinal classification can be cast as a supervised learning task, whose goal is to induce a classification model from the training data annotated with class labels. A number of supervised learning methods can be used for this purpose [22], e.g. naïve Bayes classifier, support vector machines, nearest neighbour, decision tree learning, neural networks, etc. These classification methods commonly operate under the assumption that the classes are not ordered, but can be easily adapted to make use of ordering information in class attributes to improve their performance over the naïve approach, which treats the class values as an unordered set [23]. According to the “no free lunch” theorem, any two learning algorithms are equivalent when their performance is averaged across all possible problems [24]. This theorem suggests that the choice of an appropriate algorithm should be based on its performance for the particular problem at hand and the properties of data that characterise the problem. Our choice was based on the results of cross–validation, which will be described later. First, let us discuss feature extraction, which is an important factor in terms of efficiency and classification performance.

### Feature extraction

The first problem associated with text classification is high dimensionality of the feature space. The bag-of-words representation suffers from the curse of dimensionality (or Hughes effect), where, given a fixed size of the training dataset, the predictive power of a machine learning algorithm reduces as the dimensionality increases [25]. When the ratio of the number of training instances to the number of features is low, overfitting is likely to occur. Therefore, feature selection is essential to reduce overfitting. Several feature selection methods have been proposed specifically for the problem of ordinal text classification [26]. However, these methods are based on statistical measures of informativeness such as information gain or inverse document frequency, which means that their use requires a relatively large sample size.

Alternatively, when training data are sparse, prior knowledge (i.e. any form of knowledge that may be incorporated prior to training) can be used to extract features based on their general properties without measuring their value on the training data [27]. These properties may include types of features, relationships between features, indications of feature relevance, etc. [28]. One way of incorporating prior knowledge into otherwise agnostic machine learning methods is to pre–process the training data [29]. This step consists of selecting, cleaning and transforming the original data in order to reduce noise and complexity, and increase transparency of the classification model. For example, incorporating prior knowledge into a feature vector document representation can improve text classification accuracy [30]. However, knowledge engineering itself can be fairly time and labour consuming. Ideally, prior knowledge should be re–used from existing sources or otherwise incur as little human intervention as possible. In accordance with these principles, we re–used knowledge readily available from the UMLS [21], the largest curated collection of biomedical entities, which currently covers over 3,640,132 entities and 11,757,373 names that refer to these entities. All entities are organised into a network of 133 semantic types.

#### Lexico–semantic features

To utilise the knowledge encoded in the UMLS for text mining purposes, we need to map its content onto free text. This is practically achieved by recognising NEs, i.e. names used to differentiate between entities of the same semantic type (e.g. *stiffness* and *pain* are names used to refer to specific *symptoms*), followed by normalising the representation of their meaning (e.g. *swelling* is also known as *edema* or *oedema*, all of which are associated with the same identifier). We used MetaMap, a software tool for recognising UMLS entities in biomedical text [31], to automatically identify NEs of relevant semantic types. In effect, the use of MetaMap allowed us to transform “surface” lexical features into “deep” semantic ones. For instance, when classifying answers to question Q9 about work, hobbies and exercise, we used MetaMap to identify NEs that represent instances of *daily or recreational activity* (dora), *occupation or discipline* (ocdi), *occupational activity* (ocac) and *professional or occupational group* (prog), e.g.

*Not been able to carry out my normal <* ocdi*>job</*ocdi*> at <* ocac*>work</*ocac*> due to my injury as I am a commercial <* prog*>vehicle mechanic</*prog*>. Limited <* dora*>exercise</*dora*> and <* dora*>hobbies</*dora*> I haven’t been able to play <* dora*>squash</*dora*> due to the injury and <* dora*>golf</*dora*> as I can’t twist my knee fully yet.*

References to these NEs can then be aggregated into a single semantic feature that encompasses work, hobbies and exercise. If needed, one can still differentiate between NEs of the same type. For example, when classifying answers to question Q7 in terms of overall severity, it may be useful to differentiate between various symptoms, e.g. *swelling*, *bruising*, *giving way*, *popping*, etc. by using MetaMap to map text onto the UMLS, we can aggregate multiple lexical features (e.g. *swelling*, *edema*, *oedema*, *dropsy*) into a single semantic feature, which represents the corresponding concept (e.g. *an abnormal accumulation of fluid beneath the skin or in one or more cavities of the body*). In summary, MetaMap can be used to extract semantic features at two levels of granularity (entity vs. type). In the context of machine learning, both extremes may lead to overfitting, one because of sparsity and the other because of overgeneralisation. Therefore, it may be useful to group related entities to tackle data sparsity without overgeneralising them. Consider, for instance, question Q6 about pain, where answers need to be classified on a severity scale: 1 (none) – 2 (some) – 3 (severe). Our training data contains references to *mild pain*, *severe pain*, *sharp pain*, *stabbing pain*, *intermittent pain* and *constant pain*, which are modelled as distinct entities in the UMLS. Other than grouping these entities together within the same semantic type, the UMLS does not offer any other information about their properties or relationships among them that could be useful features for ordinal text classification, e.g. that *mild pain* < *severe pain* in terms of severity or that *sharp pain* is severe. In addition, when patients discuss their pain, they often use descriptive phrases (e.g. *slight pain* or *bad pain*) rather than established terms (e.g. *mild pain* or *severe pain*). Such laymen’s terms are not represented in the UMLS and, therefore, cannot be identified in text using MetaMap

**Fig. 1:**
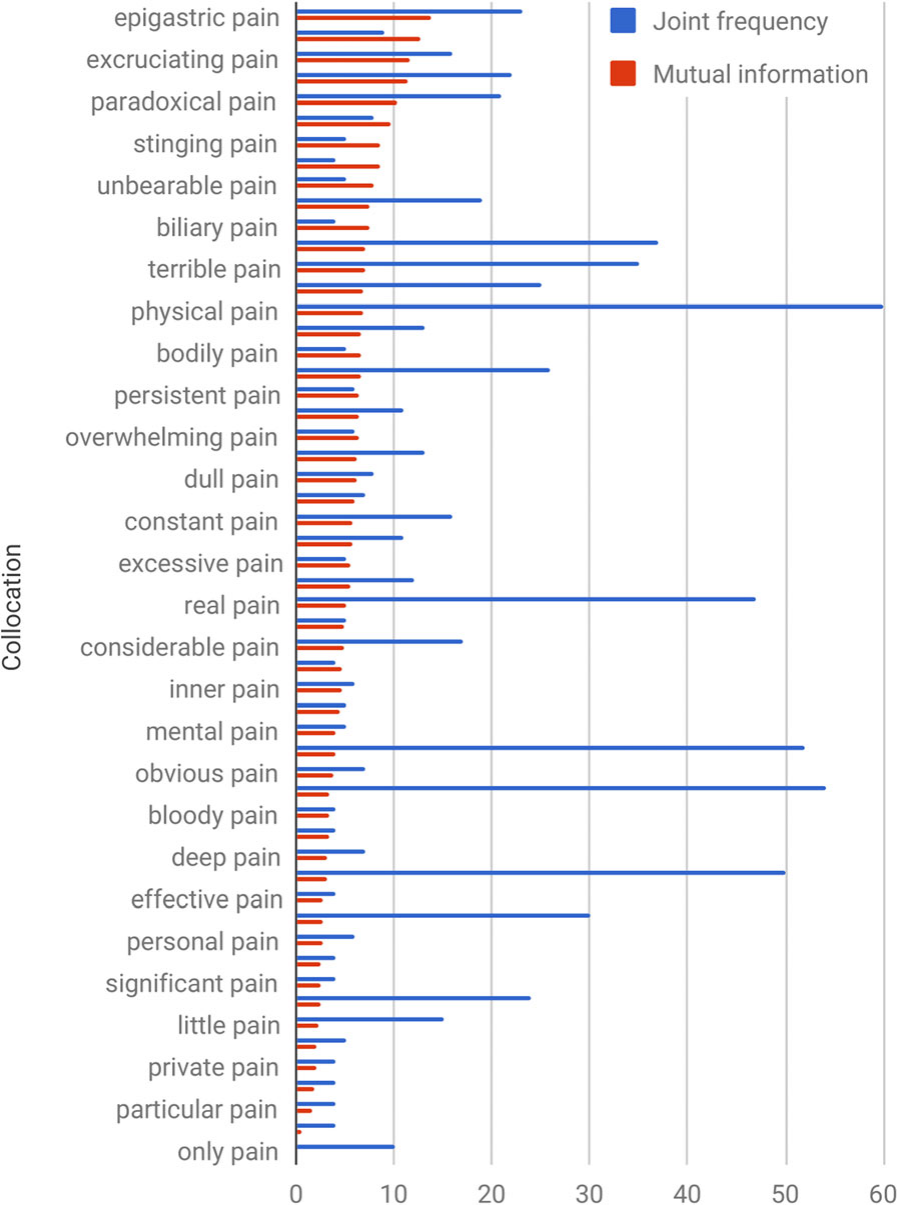
A sample of adjectives collocated with the word pain

For the purposes of ordinal text classification, it would still be useful to have some prior knowledge about such laymen’s terms, e.g. that *slight pain* < *bad pain* on a severity scale. Given that questions in our study are focusing on specific aspects of self–care, we can limit hand–crafting prior knowledge to these aspects (e.g. pain severity). We can use approaches from corpus linguistics to quickly extract relevant lexical features, and model relevant knowledge around them. For example, to systematically collect adjectives that are commonly used to modify the noun *pain*, we automatically extracted its collocations from a general corpus such as the British National Corpus (BNC) [32]. A search for collocated adjectives, where the strength of collocation was measured by mutual information, retrieved 56 items (see Fig. 1). Processing this list did not require extensive manual intervention to remove adjectives that are not applicable in the context of knee pain (e.g. *abdominal pain* or *sweet pain*) and classify the 31 remaining ones in terms of severity (e.g. *overwhelming pain* is more severe than *dull pain*). As we were only interested in laymen terminology, the manual curation did not require medical expertise.

In this manner, we efficiently supplemented prior knowledge available from the UMLS to support extraction of relevant semantic features from raw text. Note that a relatively small scale of the system in term of features and their values did not warrant automation of this approach. However, for the proposed approach to be routinely used on a large scale, a variety of text mining solutions based on distributional semantics (e.g. [33, 34]) can be used to infer relationships between different collocates automatically.

#### Negation

**Fig. 2:**
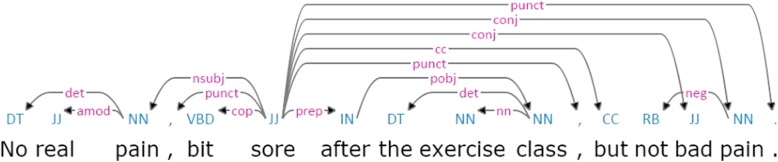
Two negated mentions of the word pain

Extraction of semantic features would not be complete without considering their context in terms of polar opposition, i.e. affirmation vs. negation [35]. Negated statements contain an additional layer of meaning and morpho–syntactic structure, which incurs an overhead in semantic processing [36]. Naturally, the processing complexity of negation poses considerable challenges in automatically detecting its scope and focus [37]. Some types of explicitly asserted negation can be recognised by using automatically extracted syntactic dependencies. For example, from the syntactic dependency parse shown in Fig. 2, it can be automatically inferred that both mentions of the word *pain* are negated.

**Fig. 3:**
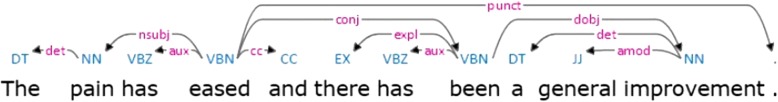
The role of verb to ease in effectively negating a nominal subject (nsubj)

On the other hand, implicit negation, whose source is pragmatic (presupposition or implicature), is not immediately accessible from the syntax and, therefore, is much harder to detect automatically. For example, the verb *to ease* is neither syntactically nor morphologically negative, but it effectively negates *pain* in the sentence given in Fig. 3.

In this study, however, we deal only with explicit syntactic negation. We used a parser distributed as part of the Stanford CoreNLP library [38] to extracted universal syntactic dependencies, which provide straightforward descriptions of grammatical relations between the words [39]. Specifically, these dependencies were used to identify negated words.

#### Sentiment polarity

**Fig. 4:**
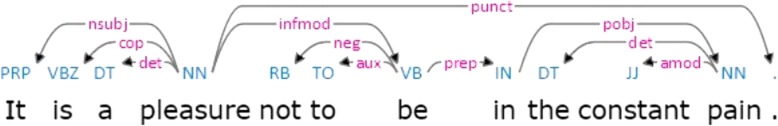
A negated mention of the word pain

In relation to questions Q5–Q7, we need to classify the severity of symptoms on a 3–point Likert scale: 1 (none) – 2 (some) – 3 (severe). For this, we need to analyse the context in which the symptoms are mentioned. Some of the features discussed thus far can be used for this purpose, e.g. negation and adjectival modifiers. For example, the two negated mentions of *pain* in Fig. 2 can be used to classify its severity as 1 (none). Similarly, the collocated adjective *intermittent* can be used to classify the severity of pain in *“Intermittent pain around the joint.”* as 2 (some), whereas the use of adjective *constant* in *“Constant pain whether sitting or standing.”* can be used to classify the pain level as 3 (severe). Combinations of different features make the analysis more complex. Consider, for example, the negated mention of *constant pain* given in Fig. 4. The analysis would require us to traverse the syntactic dependency tree to infer that the negation of the verb *to be* applies to the mention of the noun *pain*.

Alternatively, the positive sentiment of the given sentence can be used to classify the severity of pain as either 1 (none) or 2 (some), depending on the strength of the sentiment. Similarly, the negative sentiment expressed in *“My pain was unbearable and I was screaming, crying, and regretting how it happened.”* can be used to classify the pain as 3 (severe). In principle, examples of non–asserted negation given in Fig. 3 can also be handled using sentiment polarity. In our system, we used Stanford CoreNLP’s sentiment annotator [40], which uses a deep neural network approach to build up sentiment representation of a sentence on top of its grammatical structure.

### System summary

The KLOSURE system consists of two subsystems, one concerned with feature extraction and the other one concerned with classification of feature vectors. Table 1 describes the conceptual architecture of the feature extraction subsystem. Module names describe their main functionality. Specific tools used to support these functionalities are referenced in the second column. Resources used by these tools constitute the parameters of the system. Any potential improvements of the system performance will be largely confined to this area. Outputs produced by each module are combined into feature vectors. The structure of feature vectors will vary across the KLOG questions. Finally, Weka [41], a machine learning workbench, was used for classification of feature vectors. Classification is performed against a scheme described in Table 2. Specific classification methods are discussed in the following section in the context of their performance.

**Table 1:** Feature extraction subsystem

Module	Software	Resources	Output
linguistic pre-processing	Stanford Core NLP [38]	language model	POS tags, dependencies
sentiment analysis	Stanford Core NLP [40]	sentiment model	sentiment polarities
named entity recognition	MetaMap [31]	UMLS [21]	named entities, semantic types
lexicon lookup	N/A	24 lexicons	matched items

**Table 2:** Definition of ordinal classes

Question	1	2	3
Q3	worse	same	better
Q4	no	reasonably	fully
Q5–Q7	none	some	severe
Q8–Q9	not at all	somewhat	a lot
Q10	negative	neutral	positive

## Results

### Data

To collect the data, we approached individuals with knee conditions in secondary care and relevant online communities to complete the KLOG questionnaire. We collected a total of 55 responses. The size of the dataset is 7985 words, which is equivalent to 17.7 A4 pages using Arial 12 pt. and single spacing (note that this dataset is distinct from the one discussed in the Methods section). To establish the ground truth for evaluation of the KLOSURE system, all data were annotated manually. For questions Q1–Q2, annotators were asked to identify all mentions of relevant NEs and categorise them according to their types. For every mention, annotators were asked to identify the maximal extent of the string that represents an entity. Nested mentions were not annotated. The annotation task for questions Q3–Q10 was framed as ordinal text classification on a 3–point Likert scale.

**Fig. 5:**
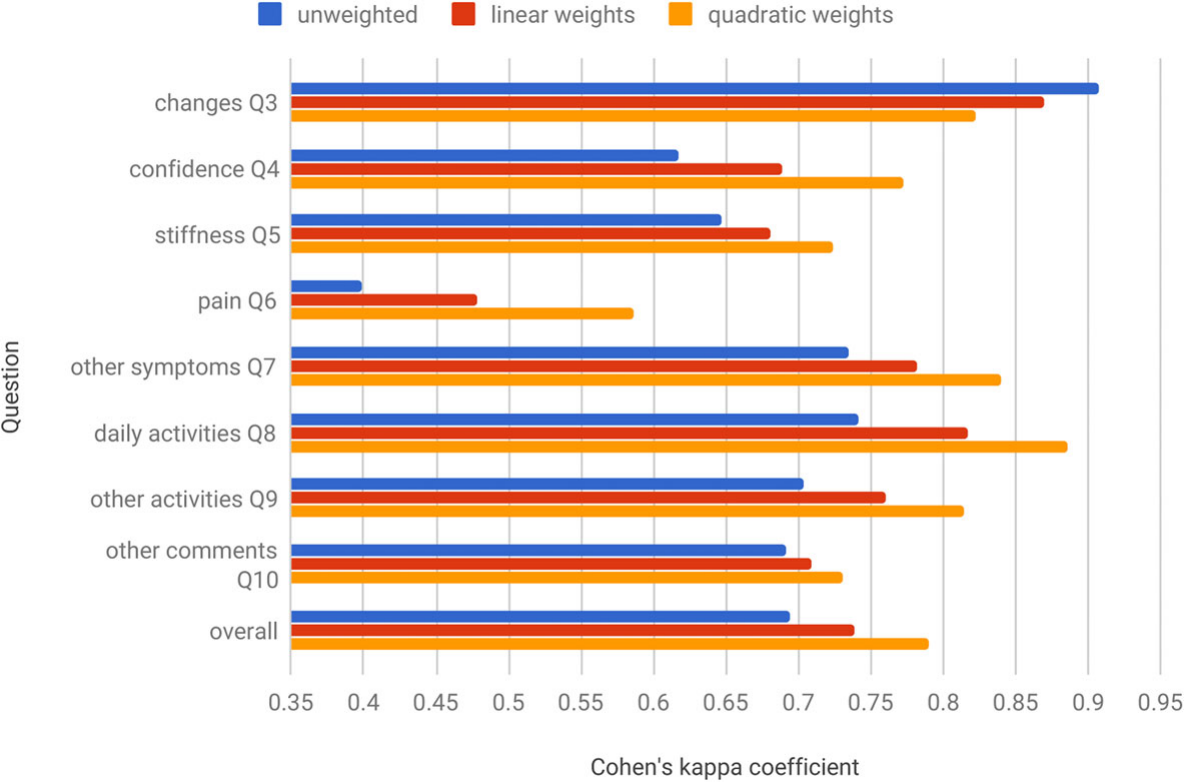
Inter-annotator agreement for questions Q3–Q10

Every response was annotated independently by two experts. Inter–annotator agreement (IAA) for annotations of NEs related to questions Q1–Q2 was calculated using Cohen’s kappa coefficient [42], whose values were 0.688 and 0.806 respectively. Cohen’s kappa coefficient treats all disagreements equally, which is not suitable when the annotation categories are ordered as they indeed are in relation to questions Q3–Q10. In such case, it is preferable to use weighted kappa coefficient, which accounts for the degree of disagreement [43]. Figure 5 provides the values of the Cohen’s kappa coefficient for questions Q3–Q10. The unweighted IAA across all 10 questions ranged from moderate (0.399) to very good (0.901). At average kappa value of 0.693 and standard deviation of 0.132, the overall IAA was found to be good [44]. The ground truth was created by the third annotator who independently resolved all disagreements.

### Evaluation

Most text mining tasks can be viewed as classification problems in which, given an instance, the system predicts its class label. For instance, a NER system in effect labels a phrase as a NE of a given type (or class). Various measures can be used to evaluate classification performance based on a confusion matrix, which contains information about actual (or known) labels and those predicted automatically by the system. Frequently used measures are that of precision (P) and recall (R), which are calculated as the following ratios [45, 46]: P = TP / (TP + FP) and R = TP / (TP + FN), where TP, FP and FN are the numbers of true positives, false positives and false negatives respectively. The given formulas are applied to calculate precision and recall for each class separately. The overall precision and recall values are calculated using weighted average, which is simply a sum of class-specific values weighted according to the number of instances within a particular class label. Systems will often be compared on how well they balance precision and recall. For this purpose, F–measure is calculated as their harmonic mean. We used these measures to evaluate the performance of the KLOSURE system.

**Fig. 6:**
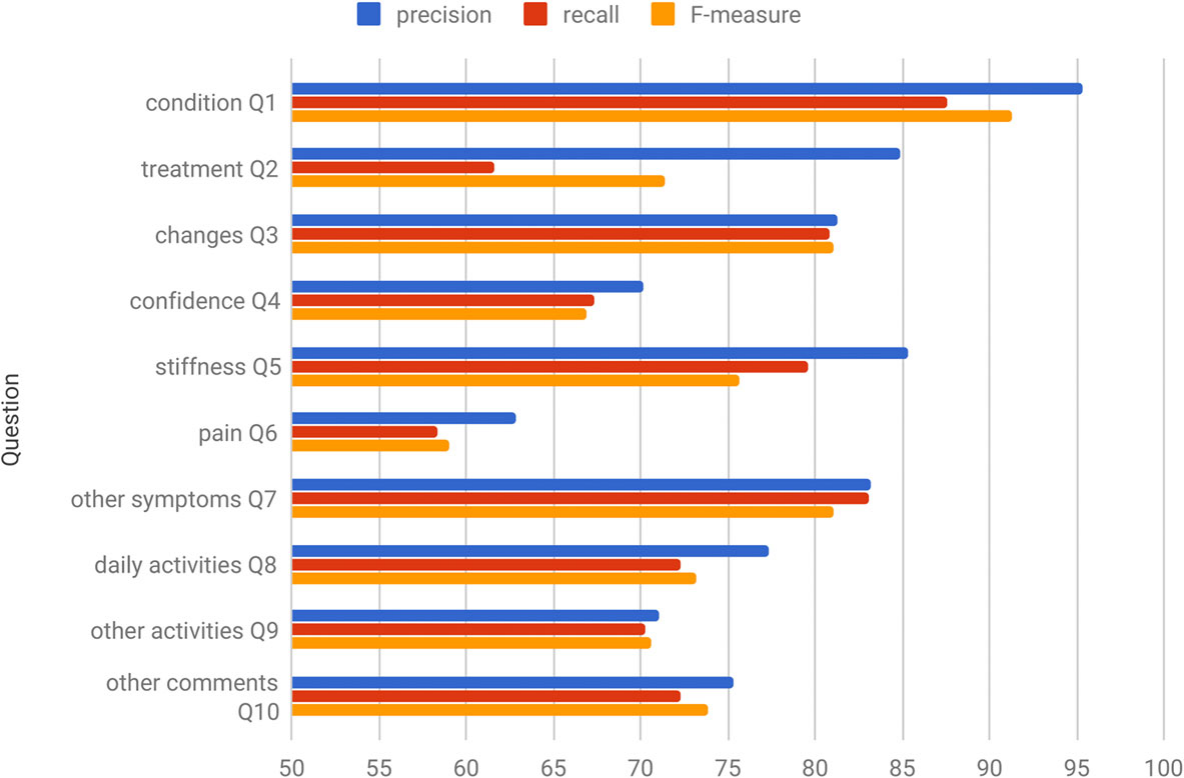
Evaluation results

The classification subsystem consists of eight modules – one classifier per question Q3–Q10. For question Q10, we re–used an existing open–source sentiment analysis tool [40]. To implement the remaining seven classifiers, we used Weka [41], a software workbench that incorporates a standard range of supervised learning methods. To systematically measure the predictive ability of a classification model for each question, we performed 10–fold cross–validation experiments. Table 3 provides the results achieved by the best performing methods. The evaluation results are also visualised in Fig. 6.

**Table 3:** Performance of the KLOSURE system

Question	Topic	Classes	Method	Features	P (%)	R (%)	F (%)
Q1	condition	3	MetaMap	N/A	95.3	87.6	91.3
Q2	treatment	4	MetaMap	N/A	84.9	61.6	71.4
Q3	changes	3	naive Bayes	8	81.3	80.8	81.0
Q4	confidence	3	best-first decision tree	8	70.1	67.3	66.9
Q5	stiffness	3	reduced error pruning tree	8	85.3	79.6	75.6
Q6	pain	3	complement naive Bayes	10	62.8	58.3	59.0
Q7	other symptoms	3	naive Bayes	5	83.2	83.0	81.0
Q8	daily activities	3	J48 pruned tree	14	77.3	72.3	73.2
Q9	other activities	3	random forest	14	71.0	70.2	70.6
Q10	other comments	3	Stanford Core NLP	N/A	75.3	72.3	73.8

## Discussion

The overall performance in terms of F–measure varied between 59.0 and 91.3% with an average of 74.4% and a standard deviation of 8.8. Given the subjective nature of pain, it is not surprising that determining the severity of pain (Q6) proved to be the most difficult classification task. Even commonly used pain rating scales are plagued with issues related to sensitivity, reproducibility, failure rate and interpretation of scores [47]. On the other hand, the factual nature of question Q1 about a diagnosed medical condition was associated with the best performance.

Similar performance would be expected in relation to question Q2 about treatments. However, recall was found to be substantially lower. The analysis of false negatives revealed two underlying causes. The first one is related to layman’s terms used by patients, e.g. *physio* and *cardio* failed to be recognised as *physiotherapy* and *cardiovascular exercises* respectively. The other one is related to under-representation of knee rehabilitation specific concepts in the UMLS, mostly specific exercises mentioned as part of the physiotherapy, e.g. *hamstring curls*. The TRAK ontology defines concepts relevant for the standard care for the rehabilitation of knee condition including a wide range of exercises [48] and can be used to supplement resources used to support NER.

Similarly, other classifiers could benefit from adding or replacing the resources used (see Table 1). For example, the sentiment analysis module uses a classification model trained on a corpus of movie reviews. Re-training the model on a corpus of patient narratives is expected to improve the performance not only in relation to question Q10 where it provides the final output, but also in relation to questions Q3–Q9 where it is used to extract sentiment-related features.

The lack of explainability has been identified as one of the major obstacles preventing widespread use of machine learning in clinical practice. We can observe that the best performing supervised classifiers tend to be of two varieties: naive Bayes learning and decision trees. The letter may improve the acceptability of the proposed text mining solution in clinical practice because they explicitly provide an explanation in the form of easily interpretable decision trees.

## Conclusion

In this paper, we demonstrated the feasibility of mining open-ended patient questionnaires. By automatically mapping free text answers onto a Likert scale, we can effectively measure the progress of rehabilitation over time. Arguably, the same can be said about closed-ended questionnaires such as KOOS. However, by spotting a negative trend, in addition to basic alert functionality, our approach offers much richer information that can be utilised to support clinical decision making. Actively listening to patients and involving them into their medical care has shown to improve health outcomes and patient satisfaction [49]. The act of writing itself may have therapeutic benefits [50]. In conclusion, we demonstrated how text mining can be used to combine the benefits of qualitative and quantitative analysis of patient experiences.

Having established the feasibility of the text mining approach, our future work will include validation of KLOSURE as a patient-reported outcome measure. This may be achieved by direct comparison of KLOSURE against KOOS. To streamline the process of data collection via KLOG, we will explore the use of voice-to-text software, which transcribes spoken words into text. In terms of productivity, text can be dictated three times as fast as typing it. State-of-the-art voice-to-text software is highly accurate, which makes it easy to use at all levels of literacy as it will automatically transcribe words that are otherwise difficult to spell. Moreover, voice commands allow for hands-free interaction, which make voice-to-text software accessible to patients with a wide range of disabilities and injuries that restrict interaction with input devices such as touchscreen display, keyboard and mouse. In summary, speed, accuracy, ease of use and accessibility make voice-to-text software a great user-friendly tool for patients to respond to open-ended questions.

## Data Availability

The dataset collected and analysed in this study is not publicly available due to data privacy. The code for feature extraction is available on demand from the corresponding author. Table 1 provides references to other publicly available software used in this study.

## Abbreviations

BNC: British National Corpus
IAA: Inter–annotator agreement
KLOSURE: KLOG measure
KOOS: Knee injury and Osteoarthritis Outcome Score
NE: Named entity
NER: Named entity recognition
P: Precision
PROMs: Patient reported outcome measures
R: Recall
TM: Text mining
TRAK: Taxonomy for RehAbilitation of Knee conditions
UMLS: Unified Medical Language System
